# Peanut oral immunotherapy: current trends in clinical trials

**DOI:** 10.1093/immadv/ltac004

**Published:** 2022-01-31

**Authors:** Simone Reinwald, Jennifer M Rolland, Robyn E O’Hehir, Menno C van Zelm

**Affiliations:** 1 Department of Immunology and Pathology, Central Clinical School, Monash University, Melbourne, VIC, Australia; 2 Allergy, Asthma and Clinical Immunology Service, Respiratory Medicine, Central Clinical School, Monash University, and Alfred Hospital, Melbourne, VIC, Australia

**Keywords:** peanut allergy, oral immunotherapy, desensitisation, biological adjuvants, clinical trials

## Abstract

Immunotherapy for allergy has been practiced for over 100 years. Low-dose repeated exposure to specific allergen extracts over several months to years can successfully induce clinical tolerance in patients with allergy to insect venoms, pollen, house dust mite, and domestic animals. Different regimens and routes for immunotherapy include subcutaneous, sublingual, oral, and intralymphatic. Food allergies have been difficult to treat in this way due to high anaphylactic potential and only recently the first immunotherapy for peanut allergy has received regulatory approval. Several clinical trials have indicated high efficacy in desensitisation of peanut-allergic individuals using oral immunotherapy, which allows for safer administration of relatively high allergen concentrations. Still, the risk of adverse events including serious allergic reactions and high anxiety levels for patients remains, demonstrating the need for further optimisation of treatment protocols. Here we discuss the design and outcomes of recent clinical trials with traditional oral immunotherapy, and consider alternative protocols and formulations for safer and more effective oral treatment strategies for peanut allergy.

## Introduction

Allergy to peanuts (*Arachis hypogaea*) is an immunoglobulin E (IgE)-mediated hypersensitivity, which tends to develop early in life and is typically lifelong with resolution in only 20% of affected children [1]. Amongst all food allergies, peanut allergy accounts for the majority of severe allergic reactions worldwide [2]. The four major peanut components of which the IgE reactivity is associated with clinically severe reactions are Ara h 1 and Ara h 3, both members of the cupin superfamily; and Ara h 2 and Ara h 6, members of the prolamin superfamily of proteins [3]. Of these four, Ara h 2 is the most specific marker allergen for severe reactions including anaphylaxis [4]. In total, 16 peanut proteins are registered as allergens to date [5]. Some of these have been characterised fully, others only partially and there may be additional, as yet unidentified protein allergens. Importantly, reactivity to some peanut components (especially Ara h 8 and 9) result from cross-reactivity to allergens in other plants and is associated with mild responses or even tolerance [6].

Allergen-specific immunotherapy (AIT) aims to increase the threshold for reactivity towards a specific allergen. The advantage of AIT is that it only modifies the immune response to the allergen of concern while not affecting the immune system at large. AIT is typically provided via regular administration of gradually increasing allergen doses. The underlying immunological mechanisms occurring over the course of AIT are subjects of current research. In general, there is a skewing of the immune response to that particular antigen away from the pro-allergenic Th2 cell activity towards an increase in Th1 cells and Interleukin (IL)-10 production by regulatory T (Treg) cells. These changes modify the B-cell response and cause a change in the IgE/IgG4 ratio [7, 8]. Allergen-specific IgG4 antibodies may inhibit IgE-mediated degranulation of target cells, which is one underlying observation in clinical tolerance [7].

Clinical trials over the last decade have evaluated various protocols of immunotherapy for peanut allergy, which typically involve the administration of gradually increasing amounts of peanut protein up to a defined maintenance dose via either the gastrointestinal tract, categorised as oral immunotherapy (OIT) or sublingual immunotherapy (SLIT), or via the skin, categorised as epicutaneous immunotherapy (EPIT) [9]. Epicutaneous and sublingual delivery of  allergens show very good tolerability but lower efficacy compared to OIT protocols [10]. Viaskin-mediated EPIT (DBV Technologies, France and USA) represents an innovative epidermal powder delivery system in a patch containing 250 μg peanut protein and has been shown to successfully increase peanut tolerance in clinical studies without evoking anaphylaxis (Table 1) [11]. Although this delivery system has a better safety record, its efficacy is modest with demonstrated benefit only in a subgroup of patients up to the age of 11 years [12].

**Table 1.** Clinical studies for peanut immunotherapy.

Category	Trial identifier	Phase	Name of trial/study	References
EPIT	NCT01170286	Phase 1	Safety of epicutaneous immunotherapy for the treatment of peanut allergy	[12]
SLIT	NCT01373242	Phase 1 Phase 2	Sublingual immunotherapy for peanut allergy and induction of tolerance (SLIT-TLC)	[13]
OIT	NCT02635776	Phase 3	Peanut allergy oral immunotherapy study of AR101 for desensitisation in children and adults (PALISADE)	[20]
	DRKS00004553	N/A	A randomised, double-blind, placebo-controlled study of oral immunotherapy in peanut-allergic children	[23]
	NCT02149719	Phase 2b/3	Boiled peanut oral immunotherapy for the treatment of peanut allergy: a pilot study (BOPI-1)	
	NCT03937726	N/A	Boiled peanut immunotherapy for the treatment of peanut allergy (BOPI-2)	
	ACTRN12617000803392	Phase 2	HYPES: peanut allergy desensitisation using sequential hypoallergenic and roasted peanuts	[25]
	NCT02163018	Phase 1	A first-in-human, randomised, double-blind, placebo controlled, single-centre study to assess the safety and tolerability of HAL-MPE1 in patients with peanut allergy	
Omalizumab + OIT	NCT02402231	Phase 2	Treatment of severe peanut allergy with Xolair (Omalizumab) and oral immunotherapy (FASTX)	[30]
	ACTRN12620001203943	Phase 4	OPAL: combining peanut oral immunotherapy and Omalizumab in adults with peanut allergy	
	NCT03881696	Phase 3	Omalizumab as monotherapy and as adjunct therapy to multi-allergen OIT in food allergic participants (OUtMATCH)	
Dupilumab + OIT	NCT03682770	Phase 2	Study in paediatric subjects with peanut allergy to evaluate efficacy and safety of Dupilumab as adjunct to AR101 (peanut oral immunotherapy)	
Abatacept + OIT	NCT04872218	Phase 2	Adjuvant treatment with abatacept to promote remission during peanut oral immunotherapy (ATARI)	
Probiotics + OIT	ACTRN12608000594325	N/A	Study of effectiveness of probiotics and peanut oral immunotherapy (OIT) in inducing desensitisation or tolerance in children with peanut allergy	[38]
	ACTRN12615001275550	N/A	Safety and efficacy of probiotic and peanut oral immunotherapy (PPOIT) for the induction of sustained unresponsiveness in children with peanut allergy.	
	ACTRN12616000322437	Phase 3	A multicentre, randomised, controlled trial evaluating the effectiveness of probiotic and peanut oral immunotherapy (PPOIT) in inducing desensitisation or tolerance in children with peanut allergy compared with oral immunotherapy (OIT) alone and with placebo	
Prebiotics + OIT	ACTRN12617000914369	N/A	Oral peanut immunotherapy with a modified dietary starch adjuvant for treatment of peanut allergy in children aged 10–16 years	
DNA vaccine	NCT03755713	Phase 1	A study to evaluate safety, tolerability and immune response in adolescents allergic to peanut after receiving intradermal administration of ASP0892 (ARA-LAMP-vax), a single multivalent peanut (Ara h 1, h 2, h 3) lysosomal associated membrane protein DNA plasmid vaccine	
Peptide immunotherapy	ACTRN12617000692336	Phase 1	Phase I trial to assess the safety and tolerability of PVX108 in peanut-allergic adults	[60]

For SLIT, peanut allergen is dissolved under the tongue for 2 min where it is taken up by oral Langerhans cells and subsequently presented to immune cells in the draining lymph nodes. The peanut SLIT extract is then swallowed, but the dose is 100–1000 times smaller than those used in peanut OIT. Results from a long-term trial in children aged 1–11 years old showed partial but clinically significant desensitisation with only rare withdrawals from adverse events and no epinephrine usage (Table 1) [13].

Among the immunotherapy protocols tested, OIT for peanut allergy has elicited a strong desensitisation effect in several trials [14]. Peanut allergen is orally administered either in natural or processed form in gradually increasing doses, with the goal of establishing tolerance to ingestion. This concept of OIT was first used by Schofield in 1908 and reclaimed application in the 1990s with the observed increasing prevalence of food allergies [15]. OIT can induce desensitisation of an allergic individual to a specific allergen, thereby providing potential protection against accidental ingestion and improving quality of life. The underlying immunological mechanisms of OIT are the subject of current research and have been associated with decreased basophil reactivity [16], increased serum IgG_4_ and IgA and initial increase followed by a decrease in serum food-specific IgE [17]. Allergen-specific memory B cells expanding during OIT point to a potential role of these cells in tolerance acquisition [18].

Although the majority of OIT-treated individuals pass the exit food challenges with an ability to tolerate increased amounts of peanut protein compared to baseline, the regimen of increasing allergen doses and performing food challenges is time-consuming, requires close supervision by medical personnel, and carries a risk of inducing a severe allergic reaction including anaphylaxis [19]. To maintain a state of desensitisation, ongoing daily intake of peanut allergen is required, because it is unknown whether clinical protection will be sustained indefinitely.

In this review, we discuss the recently approved OIT treatment PALFORZIA and emerging approaches applied in  clinical trials for peanut allergy that are aimed at improving tolerability and treatment adherence. Alternative non-IgE reactive formulations currently being investigated in clinical trials for other routes of administration are also considered for improved strategies for peanut allergy OIT.

## Oral immunotherapy advances for peanut allergy

### PALFORZIA: the first approved oral immunotherapy for peanut allergy

The international Phase 3, randomised, double-blind placebo-controlled study PALISADE (**P**eanut **AL**lergy oral **I**mmunotherapy **S**tudy of **A**R101 for **DE**sensitisation in children and adults) tested the efficacy and safety of AR101 (Aimmune Therapeutics, Inc., USA), the investigational oral biologic drug-containing peanut allergen powder (from defatted peanut flour), in peanut-allergic individuals aged 4–55 years (Table 1) [20]. This trial has recently led to the registration of the OIT product PALFORZIA^®^ in the USA and Europe [21, 22]. The primary analysis of the PALISADE trial in 496 patients aged 4–17 years indicated that 67.2% of AR101-treated patients tolerated a single highest dose of at least 600 mg of peanut protein, whereas this dose was tolerated by only 4.0% of placebo-treated patients. During the trial, systemic allergic reactions and severe adverse events were observed in 14.2% and 6% of the active group, respectively, and 3.2% and 2% of the placebo group. These adverse events led to withdrawal from the study of 11.6% of the active group and 2.4% of the placebo group. Excluding systemic allergic reactions treated during the food challenges, epinephrine was administered in 14% of the active group and 6.5% of the placebo group [20].

**Figure 1.**
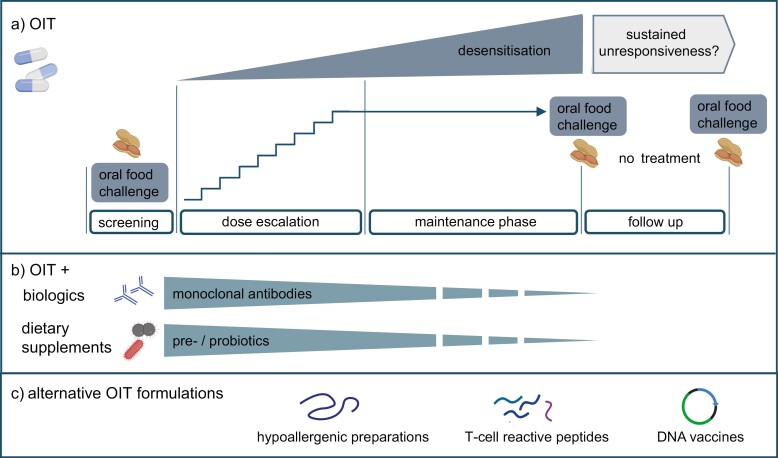
OIT strategies for specific immunotherapy for peanut allergy. (a) Typical OIT protocol. Most trials end with a DBPCFC, some with follow-up testing for sustained unresponsiveness. (b) OIT protocols with adjunct biologics or dietary supplements. Monoclonal antibodies (e.g. omalizumab, dupilumab or abatacept) or prebiotics/ probiotics are introduced either before or at the start of OIT, depending on the protocol. (c) Alternative non-IgE reactive formulations for OIT.

The treatment is now available in the USA and in Europe through a restricted program called the PALFORZIA risk evaluation and mitigation strategy (REMS) program. Healthcare providers and facilities require certification with this program prior to initiation of PALFORZIA treatment. Patients undergoing OIT with PALFORZIA do achieve an increased tolerance of peanut protein ingestion, but the protocol carries certain challenges (Fig. 1). The treatment period of approximately 6–7 months requires the patients and their families to regularly adhere to periodical medical supervision and high costs. The risk for allergic reactions persists, particularly when patients are unsupervised at home, and anxiety for these adverse events predicts a drop-out rate of ~20%, similar to that observed in the PALISADE trial [20].

## Improving the OIT protocol: timing, dosing, and allergen presentation

The risk of adverse events with peanut OIT has triggered several clinical studies with amended protocols of OIT procedures to improve the tolerability of treatment and decrease drop-out rates and anxiety levels in patients. The first approach to improve the tolerability of OIT in sensitised individuals is to slow down up-dosing and maintenance phases, escalate more gradually, and to use lower amounts of allergen. A double-blind, placebo-controlled clinical trial conducted by Blumchen *et al.* tested a peanut OIT protocol with an extended up-dosing phase of up to 14 months and a maintenance phase of 8 weeks [23]. The final open food challenge displayed desensitisation to 300 mg of peanut protein with improvement on the safety profile (Table 1). No epinephrine-requiring adverse events were encountered with fewer participant drop-outs (6.7%) recorded.

A second approach is to reduce the immunoreactivity of peanuts through thermal and chemical treatments. Enzymatic hydrolysis through heated water treatment of peanuts was identified as most effective in diminishing the allergenic potential of peanuts [24]. A hypoallergenic product generated from boiled peanuts was used in the BOPI-1 study (Boiled peanut oral immunotherapy for peanut allergy: a pilot study, NCT02149719) (Table 1). In this phase 2b/3 trial for 12 months of treatment on 47 children aged 8–16 years, participants underwent a repeat double-blind, placebo-controlled food challenge (DBPCFC) and achieved the primary outcome of desensitisation to >1.44 g peanut protein (*P* < 0.0001).

An open trial sponsored by Imperial College London is currently recruiting for the follow-up study BOPI-2 (Table 1) (NCT03937726) to demonstrate the effectiveness of boiled peanut as compared to regular peanut flour for OIT. The study aims to compare the rate of adverse events between the two interventions, will assess the immunological mechanisms involved and develop clinically useful predictors for identifying individuals likely to achieve successful desensitisation.

Australian investigators also aim to increase the safety of desensitisation and decrease the capacity to cause adverse events using hypoallergenic peanut preparations in their Phase 2 trial ACTRN12617000803392 (Table 1) [25]. A total of 66 peanut-allergic children are being treated with the hypoallergenic peanuts eaten safely (HYPES) protocol with three phases of treatment: very low allergenicity (12-h heat-treated) peanuts followed by 2-h heat-treated peanuts and finally roasted peanuts over a combined total of 52 weeks. The results are expected by the end of 2021 and will indicate if appropriate processing techniques can diminish peanut allergenicity in a clinically relevant setting.

Dutch researchers have shown a lower IgE-binding and capacity to induce allergic symptoms in peanut-allergic patients using chemically modified peanut extract (reduction and alkylation) as compared to unmodified peanut extract [26]. A clinical trial led by Danish researcher Bindslev-Jensen administered chemically modified, aluminium hydroxide adsorbed peanut extract (HAL-MPE1) subcutaneously in a Phase 1, single-centre clinical trial NCT02163018 (Table 1) and reported safety and tolerability with immunological changes in peanut-allergic patients [27]. Further trials using modified allergen extracts and formulations for OIT will determine if the tolerability of administration can be improved.

## Biologics and dietary supplements as adjuvants to improve OIT efficacy and minimise adverse events

Biologics, including drugs and immunomodulating agents or dietary supplements such as prebiotics or probiotics can help to stimulate, enhance, or modulate the immune response and therefore help to manage symptoms when given in concert with OIT.

### Omalizumab

The biological agent most utilised in the field of food allergy is Omalizumab (brand name Xolair, Genentech, Inc./Novartis Pharmaceuticals, Basel, Switzerland), an anti-IgE monoclonal antibody (mAb) currently approved for treatment of severe asthma and chronic spontaneous urticaria [28]. Omalizumab selectively binds to soluble IgE, thereby downregulating the expression of FcεRI on mast cells and basophils [28]. Omalizumab has been shown to significantly raise the tolerance threshold to food allergens and can be used as an adjunct to OIT, enabling rapid and safe escalation of food doses [29]. A Swedish study NCT02402231 (Table 1) showed that Omalizumab was an effective adjunctive therapy for initiation and rapid up-dosing of OIT for peanut allergy with very rare incidences of moderate systemic allergic reactions [30]. However, upon lowering Omalizumab intake, the adverse events from peanut OIT became more frequent [30].

Following this Swedish initiative, several studies are now trialling the utility of Omalizumab as an adjunct therapy for OIT. This includes the Australian Phase 4 trial OPAL (ACTRN12620001203943) (Table 1), a planned single-arm study to evaluate improved safety and patient acceptability over the full course of peanut OIT. OIT for multiple food allergies, including peanut, using Omalizumab induction treatment, is currently being tested in a Phase 3 multicentre placebo-controlled clinical trial (NCT03881696) (Table 1) under breakthrough designation by the US Food and Drug Administration (FDA). The designation is based on data from several clinical trials, which assessed Omalizumab’s efficacy and safety in combination with OIT for food allergens, including peanut, milk, and egg [31].

These and emerging trials will demonstrate if there is a clear benefit of Omalizumab-enabled accelerated OIT (OEAOIT) over standard OIT. A clinical advantage of Omalizumab would be a significantly decreased length of the up-dosing phase, which is the most labour-intensive and resource-consuming part of the treatment. In these trials, the dose of Omalizumab used was determined from the asthma dosage chart based on patient weight and total IgE [29]. The optimal dose to be used as an adjunct for OIT remains to be determined.

### Dupilumab

Dupilumab (brand name Dupixent, Sanofi and Regeneron Pharmaceuticals, Inc. New York, USA) is the first approved biologic by the FDA and European Medicines Agency for the treatment of atopic dermatitis (AD) with current investigations in patients with Th2-mediated bronchial asthma [32]. This therapeutic mAb is directed against the IL-4 receptor α chain (IL4Rα) and inhibits ligand binding [33]. As the IL4Rα is part of both the IL-4 and IL-13 receptors, Dupilumab interferes  with both the IL-4 and IL-13 signals. Consequently, this may decrease mast cell recruitment to the mucosa [34], thereby downregulating allergic responses and the risk of adverse events from OIT.

Regeneron Pharmaceuticals has an active trial ‘Study in paediatric subjects with peanut allergy to evaluate efficacy and safety of Dupilumab as an adjunct to AR101’ (Table 1). This Phase 2, multicentre, placebo-controlled study aims to assess whether Dupilumab improves desensitisation at the completion of the up-dosing phase of AR101 OIT with a DBPCFC at week 16 of the protocol (NCT03682770). Another objective of this trial is to evaluate the safety and tolerability of Dupilumab as an adjunct to AR101 and to assess the immunological changes in peanut-specific IgE/IgG4 ratio.

### Abatacept

Abatacept (brand name Orencia, Bristol-Myers Squibb, New York, USA) is a fusion protein of the extracellular domain of the human cytotoxic T-lymphocyte-associated antigen 4 (CTLA-4) linked to the Fc domain of human immunoglobulin 1 (IgG1). Abatacept binds to the costimulatory molecules CD80 and CD86 on antigen-presenting cells (APC), blocking interaction with CD28 on T-cells and therefore T-cell activation. Thus far, Abatacept treatment has been shown to be effective in patients with various autoinflammatory diseases [35] and belongs to a new class of medicines called biological disease-modifying anti-rheumatic drugs (DMARDs). An incidence rate of infections over long-term use (>6 months) has been reported [36], which may limit the timeframe for administration as an adjunct for OIT protocols. Currently, Abatacept is being tested as an adjuvant to OIT for peanut allergy [37]; the results of this trial may identify potential adverse effects such as increased infections. The Canadian Phase 2a, multi-centre, randomised and double-blind placebo-controlled trial ATARI (NCT04872218) (Table 1) aims to compare 24 weeks of Abatacept versus placebo as an adjuvant to peanut OIT with anticipated completion in 2023. The primary outcome is the relative change in peanut-specific/total IgE from baseline to week 24. Secondary outcomes are relative change in peanut-specific IgG4/IgE ratio at week 24 and sustained tolerance assessed between weeks 36 and 48, in addition to the highest tolerated dose of an oral food challenge at week 36.

### Pre and probiotics

The use of probiotics as a microbial adjunct therapy for OIT can enhance tolerability and decrease gastrointestinal adverse events. A double-blind placebo-controlled randomised study evaluated the effect of *Lactobacillus rhamnosus* CGMCC1.3724 in combination with peanut OIT in 62 children aged 1–10 years on the induction of sustained clinical unresponsiveness 2–5 weeks after discontinuation of treatment (Table 1) [38]. The probiotic *L. rhamnosus* CGMCC1.3724 (NCC4007) has been reported to induce Treg cells, antigen-specific IgA, and T helper (Th)1 cytokine responses [39, 40], and administration of this probiotic with peanut OIT (PPOIT) could support redirection of the peanut-specific allergic response towards tolerance. Clinical unresponsiveness two weeks post-treatment regimen was achieved in 82.1% receiving PPOIT and 3.6% receiving placebo (*P* < 0.001), associated with decreased peanut skin prick test responses, peanut-specific IgE levels, and increased peanut-specific IgG4 levels (all *P* < 0.001) [38]. The study failed to include a probiotic-only or OIT-only group limiting the ability to fully ascertain the contribution of either of the two constituents of PPOIT.

The long-term follow-up study PPOIT2 published in 2017 (Table 1) aimed to examine whether the previously reported clinical and immunological benefits of PPOIT were maintained 4 years after treatment and found that sustained unresponsiveness was maintained without the need to follow a regular pre-specified ingestion schedule [41]. These results provide a compelling argument that PPOIT induced clinical and immunological tolerance. The investigators intend to analyse the microbial composition of stool samples in future studies to examine the effects of PPOIT on the gut microbiome [41]. The Phase 3, multi-centre, 3-arm, randomised, controlled trial PPOIT-003 (ACTRN12616000322437) (Table 1) was conducted in three children’s hospitals in Australia with 200 participants aged between 1 and 10 years of age to evaluate the effectiveness of PPOIT in inducing sustained unresponsiveness compared with peanut OIT alone and with placebo, with complete data analysis anticipated by the end of 2021.

Another Australian interventional study (ACTRN12617000914369) (Table 1) will assess clinical tolerance and sustained unresponsiveness after administration of a modified dietary fibre (butyrylated high amylose maize starch) in conjunction with a daily dose of roasted peanut powder in peanut-allergic children. This prebiotic adjunct strategy aims to enhance beneficial gut bacteria and increase the levels of the short-chain fatty acid butyrate, which may promote a non-allergic environment [42, 43].

#### Alternative formulations for OIT for peanut allergy

In addition to whole peanut protein preparations and adjunctive therapeutics, alternative allergen forms for immunotherapy are being researched and trialled to overcome the  associated adverse events of exposing the gastrointestinal tract to whole allergenic protein. DNA vaccines and peptide therapeutics are under investigation for other routes of administration and could be considered for OIT. Hypoallergenic preparations also offer an alternative. These interventions potentially could decrease treatment duration, the need for strict medical supervision and may lead to higher patient adherence.

### DNA vaccines

A novel immunotherapeutic approach utilises intradermal administration of plasmid DNA encoding peanut allergens and is currently being tested for safety, tolerability, and immune response in a Phase 1 trial (NCT03755713) (Table 1) in 20 patients aged 12–17 years. The ARA-LAMP-vax (Immunomic Therapeutics Inc., Pennsylvania, USA) comprises one DNA plasmid encoding the major peanut allergens Ara h 1, Ara h 2, and Ara h 3, encoded as fusion proteins with lysosomal associated membrane protein (LAMP) [44]. Immunomic therapeutics hypothesise that this plasmid DNA vaccination is a strategy to activate natural killer (NK) cells that produce interferon-gamma (IFNγ) [45], influencing skewing of Th cell responses. The LAMP technology diverts the synthesised Ara h protein products of the vaccine directly to the lysosome in the dendritic cells, making them readily available to form antigen-MHC-II complexes. Once on the cell surface, these MHC-II complexes can directly interact with Th cells and in the presence of IFNγ, lead to the production of antibodies and Th1 cytokines [45, 46]. Trial completion is anticipated by the end of 2021, and results will inform treatment safety and whether the immune response could be redirected from a Th2/IgE allergen-specific response to a Th1/IgG response.

Vaccination with plasmid DNA will need further proof against apprehensions about the safety of this vaccine type, categorised as gene therapy. Issues have been raised due to the hypothetical risk of integration of the vaccine itself into the genome or long-term persistence of the administered plasmid DNA that could lead to triggering the production of anti-DNA antibodies [47]. These aspects will require further  consideration. A potential alternative methodology would be an mRNA-based vaccine, without the capacity to integrate into the host genome, as has been extremely successful in vaccines against SARS-CoV-2 [48, 49].

### Modified peanut allergen preparations and other adjuvants

Other vaccination approaches are currently undergoing pre-clinical testing in animal studies. A study performed by Storni et al. used peanut-sensitised mice to demonstrate a protective effect of vaccination with extracts of roasted peanut or the single allergens Ara h 1 or Ara h 2 coupled to immunologically optimised Cucumber Mosaic Virus-derived virus-like particles (CuMVtt) [50]. Modified hypoallergenic preparations are proving advantageous for other allergies and could also be considered for the safe treatment of  peanut allergy [51]. In particular, potential induction of eosinophilic oesophagitis by OIT could be averted by using hypoallergenic preparations [52].

The alteration of allergenic protein structure and function influences immunoreactivity and allergenicity [24]. Amongst these approaches, the most advantageous for diminishing the allergenic potential of peanuts is enzymatic hydrolysis [53]. Further investigations have shown reduced allergenicity using a combination of enzymatic hydrolysis followed by, or in conjunction with, roasting [54], high pressure and heat [55], or ultrasound with enzymatic treatment [56], collectively called Hurdle technology.

### Peptide immunotherapy

An alternative approach to prevent IgE-mediated adverse events is to utilise T-cell reactive peptide fragments of the allergen that are not recognised by antibodies [57]. Australian researchers have designed a peanut peptide product designed to induce tolerance to peanuts and decrease the risk of severe allergic reactions upon accidental exposure [58, 59]. Biotechnology company Aravax Pty Ltd (Melbourne, Australia) conducted a double-blind placebo-controlled Phase 1 trial (ACTRN12617000692336) (Table 1) in peanut-allergic adults to test PVX108, a mixture of synthetic peptides that represent immunodominant T-cell epitopes of Ara h 1 and Ara h 2 [58, 59]. The Phase 1 study comprised two phases, the first of which assessed single, ascending doses (0.05–150 nmol) of PVX108 and enrolled eight patient cohorts, randomised to receive PVX108 or placebo by intradermal injection with staggered successive cohort dosing such that the eighth cohort received the highest dose. In the second phase, 18 additional subjects were randomised to receive six injections of 150 nmol PVX108 over a 16-week period. The Phase 1 study demonstrated that PVX108 had a highly favourable safety profile, even for patients with severe peanut allergies. No treatment-related reactions were deemed to be of clinical concern by the Safety Review Committee and no ‘allergic’ adverse events were noted. In addition, there was a demonstrated lack of *in vitro* basophil reactivity to PVX108, providing further support for an improved safety profile of PVX108 over whole allergen immunotherapies [60].

## Conclusions

OIT enables the administration of much higher doses of allergen with greater safety than other routes of administration of AIT. The recently approved peanut OIT product PALFORZIA represents a promising treatment option for peanut-allergic patients to increase the threshold of clinical tolerance for protection from accidental ingestion and possibly achieving sustained unresponsiveness. Clinical data collection of healthcare providers in the REMS program will help ongoing research in assessing the effectiveness of PALFORZIA in the real-world setting and guide future clinical trial design for OIT.

This and other new strategies to enhance the safety and efficacy of immunotherapy for peanut allergy are currently under clinical exploration. Promising trends amongst different protocols and methods to improve OIT success with decreased adverse events are to alter OIT protocols regarding timing and dosing: a lower allergen delivery dose and a slow, delayed escalation rate in OIT treatment seem approachable in the near future.

Adjunctive therapy with biologics in parallel to OIT protocols may help to mitigate risk and improve tolerability, especially in highly allergic individuals. Many trials using the biologic Omalizumab as adjunctive treatment in OIT have shown a raised threshold tolerance dose of OIT treatment, thereby reducing the risk of severe adverse reactions. Other biologics such as Abatacept are not optimal for extended use, which limits their application in OIT protocols. Dietary supplements such as probiotics have been shown to improve treatment tolerability with decreased adverse effects over longer periods of time, presenting a cost-effective adjunct treatment with OIT.

Alternative formulations that circumvent exposure of the gastrointestinal tract to IgE-reactive, whole allergenic proteins, such as peptides, DNA vaccines, and other hypoallergenic preparations, could present a safer, more cost-effective approach for OIT for peanut-allergic individuals.

More studies are needed to fully elucidate the best protocol to achieve sustained unresponsiveness. Most current clinical trials include objective laboratory biomarker analysis in patients undergoing OIT protocols [8]. The rapid developments in identifying new biomarkers for early prediction of treatment success may facilitate understanding of underlying immunological mechanisms and possibly stratify those patients at higher risk for adverse reactions [61].

## Data Availability

There are no novel data generated associated with this review article.

## Abbreviations

AD: Atopic dermatitis
AIT: Allergen-specific immunotherapy
CTLA-4: Cytotoxic T-lymphocyte antigen 4
DBPCFC: Double-blind: placebo-controlled food challenge
DMARDs: Disease-modifying anti-rheumatic drugs
EPIT: Epicutaneous immunotherapy
IFN: Interferon
IL: Interleukin
LAMP: Lysosomal associated membrane protein
mAb: Monoclonal antibody
OEAOIT: Omalizumab-enabled accelerated oral immunotherapy
OIT: Oral immunotherapy
PPOIT: Probiotic with peanut OIT
REMS: Risk evaluation and mitigation strategy
SCFA: Short-chain fatty acids
SLIT: Sublingual immunotherapy
Th cell: T helper cell
Treg cell: T regulatory cell
